# Early-life sexual segregation: ontogeny of isotopic niche differentiation in the Antarctic fur seal

**DOI:** 10.1038/srep33211

**Published:** 2016-09-13

**Authors:** L. Kernaléguen, J. P. Y. Arnould, C. Guinet, B. Cazelles, P. Richard, Y. Cherel

**Affiliations:** 1 Deakin University, Geelong, Australia School of Life and Environmental Sciences (Burwood Campus); 2 Centre d’Etudes Biologiques de Chizé, UMR 7372 du CNRS-Université de La Rochelle, 79360, Villiers-en-Bois, France; 3 Eco-Evolutionary Mathematics, IBENS, Ecole Normale Supérieure, Paris, France; 4 UMMISCO, Université Pierre and Marie Curie et Institut de Recherche pour le Développement, Bondy, France; 5 Littoral Environnement et Sociétés (LIENSs), UMR 7266 du CNRS-Université de la Rochelle, 2 rue Olympe de Gouges, 17000 La Rochelle, France

## Abstract

Investigating the ontogeny of niche differentiation enables to determine at which life-stages sexual segregation arises, providing insights into the main factors driving resource partitioning. We investigated the ontogeny of foraging ecology in Antarctic fur seals (*Arctocephalus gazella*), a highly dimorphic species with contrasting breeding strategies between sexes. Sequential δ^13^C and δ^15^N values of whiskers provided a longitudinal proxy of the foraging niche throughout the whole life of seals, from weaning, when size dimorphism is minimal to the age of 5. Females exhibited an early-life ontogenetic shift, from a total segregation during their first year at-sea, to a similar isotopic niche as breeding females as early as age 2. In contrast, males showed a progressive change in isotopic niche throughout their development such that 5-year-old males did not share the same niche as territorial bulls. Interestingly, males and females segregated straight after weaning with males appearing to feed in more southerly habitats than females. This spatial segregation was of similar amplitude as observed in breeding adults and was maintained throughout development. Such early-life niche differentiation is an unusual pattern and indicates size dimorphism and breeding constraints do not directly drive sexual segregation contrary to what has been assumed in otariid seals.

In many species, the ecology of males and females differ greatly, the two sexes occupying distinct niches and playing different roles and functions in their ecosystem to the extent that they may be regarded as separate species^1^,^2^. While sexual segregation is likely to be caused by a combination of factors (e.g. size dimorphism, activity budget, physiological requirements), it is important to disentangle the effect of each parameter to understand their respective role. However, differentiating the influence of various factors is often difficult in empirical studies. In that context, studying the ontogeny of sexual segregation provides a good opportunity to understand underlying mechanisms explaining sex-specific variability^3^,^4^. Indeed, most organisms exhibit ontogenetic shifts in their resource-use associated with changes in life-history requirements (e.g. growth, reproduction, social interactions)^5^,^6^. Hence, the comparison of the ecological niche of males and females as they age enables us to reveal when segregation occurs in the species development and provides insights into the major factors driving sexual niche differentiation. For instance, in dimorphic species, does niche divergence mirror size divergence? Does sexual segregation arise only when individuals reach sexual maturity?

When the two sexes are characterised by contrasted life-history traits, comparing their respective ontogenetic trajectories enables us to study the factors influencing an individual to change its resource-use as it ages. However, only a few studies have investigated the development of sexual segregation^7^,^8^,^9^, with juveniles of both sexes being pooled in a single group in most studies investigating the effect of age and sex on niche partitioning. Furthermore, when comparing the ecological niche of males and females of different age groups, cross-population studies do not account for the inter- and intra-individual variability which is increasingly recognised as accounting for a major component of population variability (but see^3^,^10^ for examples of longitudinal studies).

Adult Antarctic fur seals (*Arctocephalus gazella*) exhibit strong niche differentiation between the sexes^11^,^12^. Males and females segregate spatially, with males typically foraging deeper and further away from the breeding colonies than females^11^,^12^,^13^. Furthermore, when feeding in the same water masses, they differ in their diet, with males feeding at a higher trophic level than their female counterparts^11^,^13^. As for many other otariid species (fur seals and sea lions), sexual size dimorphism and variation in breeding constraints have been proposed as the main drivers explaining niche variation. Indeed, the Antarctic fur seal is a highly dimorphic species, with territorial bulls being up to four times the mass of the largest females^14^. While the two sexes reach sexual maturity on average by the age of 3–5, males do not start to reproduce before they are 6–10, when they are large enough to hold a territory^15^. They do not provide any parental care and can disperse after the mating season. In contrast, breeding females suckle their single pup on land for four months. As females need to regularly come back to the breeding colony, their at-sea foraging grounds are spatially restricted during the pup-rearing period.

Whiskers of Antarctic fur seals are a keratinous (*i.e.* metabolically inert) tissue that grows continuously at a constant rate^11^,^16^. It has been shown on subantarctic (*A. tropicalis*, n = 2)^11^ and South American (*A. australis*, n = 3)^17^ fur seals that serially sampled whiskers could potentially record the isotopic niche over the entire life of young seals (<5 year-old) at a fine scale; with δ^13^C and δ^15^N values used as proxies of the foraging habitat and trophic level, respectively. In the present study, the isotopic signature of male and female Antarctic fur seal whiskers were analysed to document the foraging ecology of seals during their early-life, and compare their ontogenetic trajectories from weaning, when size dimorphism is minimal <15%^18^, to age 5, when individuals have reached sexual maturity. We expected that:

1. In relation to their younger age at breeding, females would reach the isotopic niche of breeding females at a younger age than males would reach the isotopic niche of territorial bulls;
2. The isotopic niche of males and females would progressively diverge throughout ontogeny, from identical niches in the first months at-sea to the well-defined and distinct niches of breeding adults, in parallel with the progressive increase in sexual size dimorphism.

## Results

### Age determination

**Figure 1:**
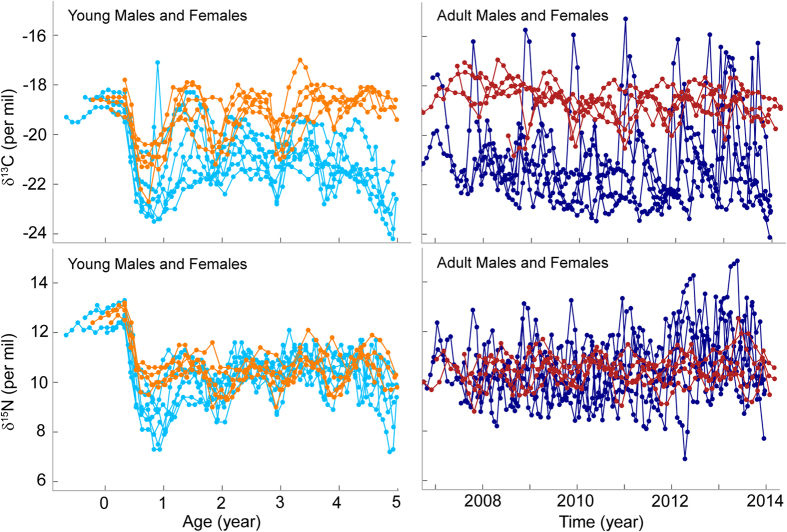
Whisker δ^13^C and δ^15^N values of males (light blue) and females (orange) from lactation to the age of 5, and of full-size territorial males (dark blue) and adult breeding females (dark red), over seven years.

Seven young male and five young female whiskers exhibited high δ^15^N values at the tip, followed by an abrupt drop characteristic of the lactation and weaning periods, respectively (Fig. 1, Supplementary Fig. S1). These whiskers measured 170 ± 38 and 134 ± 15 mm, respectively, while full-size breeding male and female whiskers measured 261 ± 68 and 166 ± 21 mm, respectively, corresponding to a total of 1,333 isotopic samples analysed.

Wavelet analyses indicated that all seals exhibited significant periodic oscillations along the length of their whisker in either δ^13^C or δ^15^N values: 68% along the entire length (80% of the cone of influence^11^) and 32% along only a part of the whisker. Importantly, when periodicity was statistically detected, the period of cycles was constant along the length of every whisker, including the whisker of younger individuals. While Rea *et al*.^19^ have identified age specific whisker growth rates in Steller sea lions (*Eumetopias jubatus*), this result supports the assumption of a constant whisker growth rate from weaning throughout the entire development of Antarctic fur seals. Although we cannot preclude a variation in growth rate in the portion of whisker that did not exhibit a statistically significant periodicity (for 4 young seal whiskers), cycles were detected on 62 or 68% of the cone of influence corresponding to the early life of seals for 2 of these individuals, indicating a constant growth rate from weaning until the age of 3. Furthermore, assuming the isotopic cycles correspond to the annual cycle of seals^11^,^19^,^20^, the wavelet analysis estimation of the age of the tagged 5-year-old male was 4.9 (Supplementary Fig. S1), confirming the constant growth rate assumption, and the utilisation of the annual isotopic cycles to estimate seals age.

All younger seals (whose whisker recorded their entire life) were either five or six years old. Whiskers from older breeding males and females recorded on average 6.6 ± 0.8 and 7.3 ± 2.1 y, respectively. The corresponding whisker growth rates were 0.09 ± 0.03 and 0.07 ± 0.01 mm·d^−1^ for younger males and females, and 0.11 ± 0.03 and 0.06 ± 0.01 mm·d^−1^ for older adult males and females, respectively.

### Ontogeny of female and male isotopic niche

**Figure 2:**
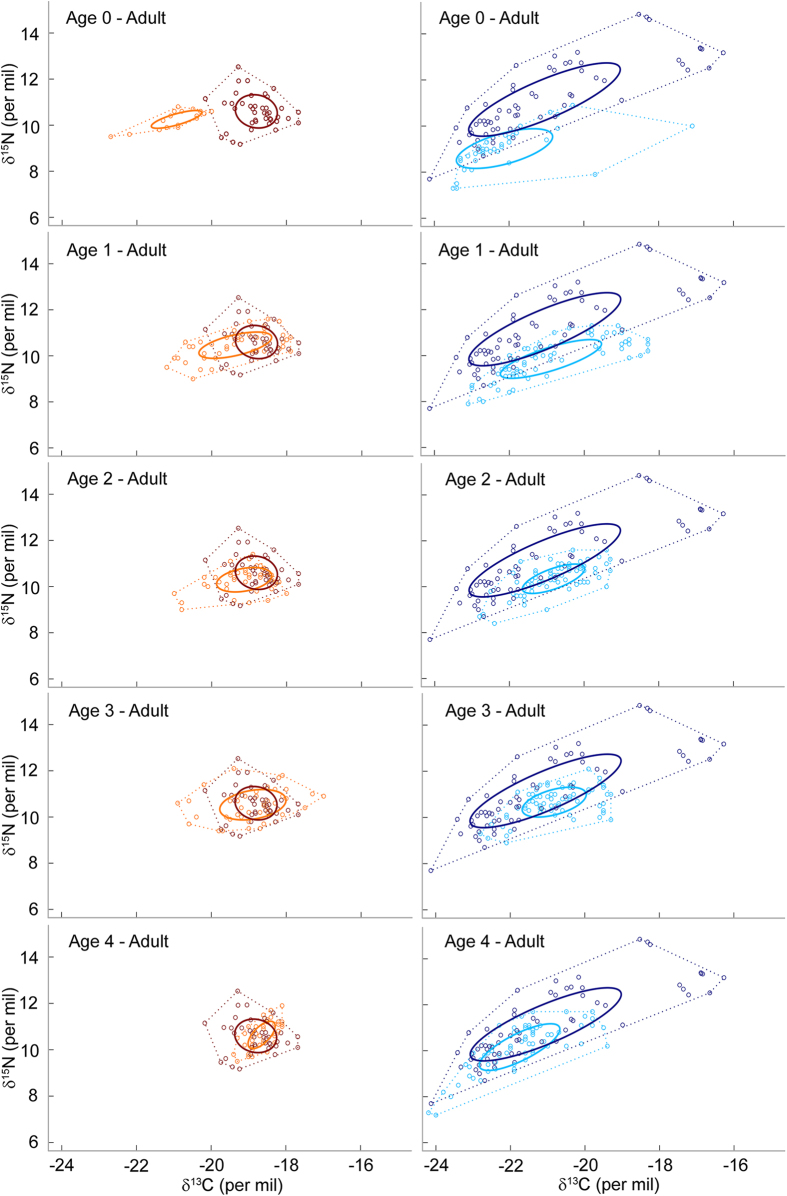
Ontogenetic changes in the isotopic niche of young female (orange) and male (light blue) Antarctic fur seals during the first 5 years of life. The isotopic niche of full-size breeding females (dark red) and males (dark blue) are displayed for a better comparison. Each dot corresponds to a whisker segment, and solid lines to the SEAc of each group (on which percentage of niche overlap is calculated). Convex hull areas are represented in dotted lines, as a reference.

The isotopic niche of females showed a step-change as seals aged (Fig. 2). Females showed a complete segregation at age 0, with no overlap in their core isotopic niche with all other age-classes, including breeding adults (i.e. no overlap between the standard ellipse area corrected for unbalanced sample sizes (SEAc) at age 0 and the SEAc at other ages, Supplementary Table S1). Following that first year at-sea, the isotopic niche of individuals greatly overlapped throughout their development. Ontogenetic variation in female isotopic niche occurred exclusively along the δ^13^C axis. Females had significantly lower δ^13^C values at age 0 than the following years, while individuals showed similar δ^15^N data at all age-classes (Fig. 2, Supplementary Table S2).

Unlike females, males exhibited a progressive change in isotopic niche through time (Fig. 2). The overlap was minimal between territorial bulls and young males at age 0 and 1 (0 and 2% overlap, respectively), and increased after age 2 (41, 75 and 74%, Supplementary Table S3). Variation in male isotopic niche occurred both along the δ^13^C and δ^15^N axes (Fig. 2, Supplementary Table S4). In particular, δ^15^N increased with age until age 3. Territorial males encompassed a greater range of δ^13^C and δ^15^N values, which were on average higher than isotopic values of younger males (Supplementary Table S4).

### Ontogeny of sexual isotopic niche segregation

During lactation, male and female pups exhibited similar δ^13^C and δ^15^N values (δ^13^C: −18.9 ± 0.4‰ and −18.5 ± 0.6‰, respectively, t_6,4_ = −1.14, P = 0.29, δ^15^N: 12.7 ± 0.4‰ and 12.8 ± 0.3‰, respectively, t_6,4_ = −0.91, P = 0.39). At weaning, all seals of both sexes exhibited an abrupt drop in their δ^13^C and δ^15^N values (Fig. 1). However, the drop was more pronounced in males, such that males had lower values than females during their first year (δ^13^C: −22.2 ± 1.3‰ and −21.0 ± 0.8‰, δ^15^N: 9.0 ± 0.9‰ and 10.2 ± 0.4‰, respectively).

**Figure 3:**
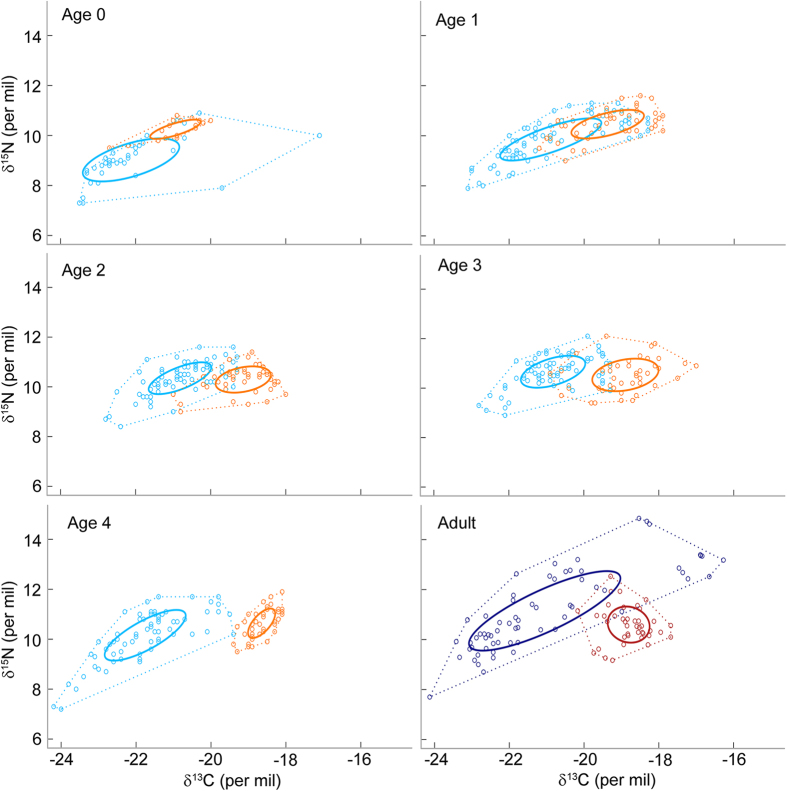
Sexual segregation in isotopic niche at each class-age. Young males and females are represented in light blue and orange, respectively, and full-size breeding males and females are represented in dark blue and dark red, respectively. Each dot corresponds to a whisker segment, and solid lines correspond to the SEAc of each group (on which percentage of niche overlap is calculated). Convex hull areas are represented in dotted lines, as a reference.

Males and females of similar age-class exhibited a complete niche differentiation throughout their development, except for age 1 (when seals are between 1 and 2 year-old) where the overlap was 18 and 29% for males and females, respectively (Fig. 3, Table 1). While the two sexes varied in both δ^13^C and δ^15^N values at age 0 and to a lesser extent age 1, sexual segregation occurred exclusively along the δ^13^C axis later in seals’ development, with males exhibiting consistently lower δ^13^C values than females (Table 2). Males occupied a larger isotopic space than females at early stages (ages 0 and 1), and at age 4 (Table 1). However, individual niches were similar in size (Supplementary Table S5), denoting either (i) smaller sample sizes at the individual level (lack of statistical power due to a smaller number of whisker segments per individual) and/or (ii) greater inter-individual variation in isotopic niche between males. Difference in sample size between males and females is unlikely to explain this pattern as the standard ellipse areas corrected for unbalanced sample sizes (SEAc, Bayesian SIBER analyses) allow robust comparison among data sets of contrasted sampling size^21^.

Full-size breeding males and females exhibited a complete segregation in their respective isotopic niche (Fig. 2). Variation occurred along two axes: (i) males had depleted ^13^C values compared to females, but no difference in average δ^15^N values (Table 2); and (ii) territorial males showed a much wider isotopic niche than breeding females, both at the population and individual levels (4.5 and 6.9 times larger, respectively, Fig. 3).

## Discussion

The whisker stable isotope patterns observed in the present study depict the early-life of individual Antarctic fur seals, documenting the ontogenetic patterns of habitat and resource-use and revealing new insights into cryptic stages of a seal’s life. While results of this study are based on a relatively small sample size (7 females and 5 males, but representing a total of 1,333 isotopic samples), the sexual niche differentiation of Antarctic fur seals appears to be a rather abrupt process occurring early after weaning being, thus, mostly decoupled from sexual size dimorphism. Foraging ecology was strongly linked to sex-specific life-history traits. In females, maturing and breeding earlier, young individuals shared the isotopic (ecological) niche of adults at 1–2 years-old. In males, that mature and breed later in life, the shift in isotopic niche to that of adults was more progressive. There was a temporal decoupling between the isotopic niche at sexual maturity and reproduction, with the mature (5-years-old) individuals occupying only a part of the isotopic niche of the territorial bulls. Adult males and females are characterised by dramatic differences in life-history traits. Hence, comparing the ontogenetic trajectories of individuals sampled the same year (i.e. experiencing the same environmental conditions) enabled us to investigate the driving forces shaping sexual segregation and the factors that influence a seal to change its foraging ecology as it ages.

### Ontogeny of female isotopic niche

Female Antarctic fur seals exhibited a rapid change in their isotopic niche as they aged, from a complete segregation with adults during their first year at-sea, to a similar isotopic niche as soon as they reached the age of 2. This early life ontogenic shift occurred exclusively along the δ^13^C axis. Weaning was characterised in every sampled females by an abrupt drop in carbon values, such that there was no overlap between young of the year (YOY) and adults’ δ^13^C values. In the Southern Ocean, δ^15^N baseline is relatively stable from Antarctica to the subtropical front^22^. In contrast, lower latitude plankton food bases are enriched in ^13^C relative to higher latitude waters^23^, a latitudinal gradient also reflected in top order predators^22^,^24^. While breeding females are known to stay around the breeding colony^25^,^26^, results suggest the sampled females travelled south of Kerguelen at weaning, and foraged exclusively in Antarctic waters south of the Polar Front. Such spatial segregation could potentially denote an early life exploration phase in females. Alternatively, niche differentiation could be the result of social exclusion, YOY exploiting sub-optimal foraging areas as they are excluded from higher quality grounds by older dominant individuals^27^,^28^.

Surprisingly, the sampled females exhibited no variation in δ^15^N values as they aged. Newly weaned pups and breeding adults present significative physiological differences (e.g. different growth rates) that might be reflected, to some extent, in their nitrogen isotopic values^29^. While such isotopic variation is poorly documented, results suggest YOY females fed at the same trophic level as adult breeding females. Previous diet studies have shown that breeding females feed almost exclusively on myctophid fish (up to 94% of their diet)^13^,^26^,^30^. Whisker δ^15^N values are consistent with these studies and suggest that females in this study primarily forage on myctophids throughout their whole life (although potentially on different species depending on the foraging area). In many species, individuals show an ontogenetic variation in their diet, be it a sudden shift or a progressive change. Temporal change in feeding behaviour is often observed when specific foraging skills are required to capture prey^31^, prey size is directly linked to predator’s size^32^, and/or individuals differ in their food requirements depending on their breeding status^6^ or growth rate^3^. However, in the present study, small and naïve females fed at the same trophic level, and most probably upon the same prey, just after the weaning period compared to when they became older, larger breeding females.

### Ontogeny of male isotopic niche

Unlike females which displayed a rather abrupt early life ontogenetic shift, males exhibited a more progressive change in their isotopic niche with age. YOY males exhibited very low δ^13^C and δ^15^N values straight after weaning, corresponding to the lowest extent of the isotopic niche of territorial bulls. This most probably indicates the sampled males travelled south after weaning, up to the southern foraging grounds of breeding bulls in Antarctic waters where they fed at least partly on low-trophic level prey, likely Antarctic krill (*Euphausia superba*), as indicated by their relatively low δ^15^N values^11^,^16^. Following their first year at-sea, young males foraged in northern areas where krill is not as abundant and fed at the same trophic level as females. Young males’ δ^15^N values increased progressively with age, and 5-year-old males exhibited lower isotopic values than breeding bulls when foraging within similar water masses, indicating that males feed on higher trophic level as they get older, larger and more experienced.

While females occupied a similar isotopic niche as adults as soon as the age of 2, 5-year-old males did not share the same foraging niche as full-size territorial bulls. Adults exhibited (i) higher δ^15^N values when feeding in the same water masses, and (ii) an extreme range of δ^13^C and δ^15^N values, encompassing young males’ isotopic niche. Indeed, territorial bulls exhibited synchronous peaks in δ^13^C and δ^15^N values. These atypical high isotopic values were not present in young males and were observed exclusively during the most recent years in breeding adults’ whiskers (Supplementary Fig. S1), suggesting peaks are associated with reproduction. While physiological variation (e.g. fasting) or the latitudinal gradient are unlikely to explain alone these extreme values, isotopic results are more likely to depict a benthic foraging behaviour on the continental shelf around the breeding colony, with benthic consumers consistently showing high δ^13^C and δ^15^N values^22^,^33^. Accordingly, two tracking studies on full-size male Antarctic fur seals have shown they displayed benthic dives on the continental shelf during the mating season^12^,^34^. In this study, benthic foraging behaviour appears late in males’ development, most probably when they start holding territories.

Males and females showed contrasted ontogenetic trajectories which mirror their respective growth and reproductive status development. Indeed, females achieve 90% of their growth by the age of 4^15^, and the majority of them first give birth at 3 or 4^35^. Hence, the first females to reproduce are gestating at 2, and meet most of the nutritional and energetic requirements of full-size breeding adults with which they share the same isotopic niche. In contrast, males grow constantly until the age of 7^15^, when the dominant individuals start to hold territories and reproduce. As they dramatically increase in body size, they vary in their energetic requirements and ability to catch larger prey. Furthermore, body size is directly related to an individual’s oxygen stores which determine its aerobic dive limit^36^ and, thus, the extent of the water column that can be efficiently exploited. In particular, males need to have a sufficient body size in order to have the physiological requirements to reach the seabed over the Kerguelen shelf.

### Ontogeny of sexual segregation

Comparison of the isotopic niche of full-size males and females confirmed the presence of strong sexual segregation in dimorphic breeding adults of Antarctic fur seals at Kerguelen, as previously described elsewhere^11^,^12^,^13^. In particular, males exhibited lower δ^13^C values suggesting they foraged in Antarctic waters most of the breeding cycle while females remained closer to the breeding colony. Sexual size dimorphism and contrasting breeding strategies have often been proposed as the main factors explaining sexual segregation in Antarctic fur seals^11^,^12^,^13^, and other otariids^37^,^38^,^39^. This suggests males and females should exhibit a similar foraging niche during their early development, and diverge in their feeding behaviour as size dimorphism increases and/or seals start to reproduce^3^,^40^.

Interestingly however, males and females showed a complete segregation in their isotopic niche straight after weaning, when size dimorphism is minimal, between 0 and 15%^18^, and seals do not face any breeding constraints. Variation in δ^13^C values was of similar amplitude as observed in breeding adults and niche differentiation was maintained throughout seals’ development until they reached sexual maturity. Variation in foraging grounds between weaned males and females has previously been reported in Antarctic fur seals breeding at Bird Island^41^. However, while males travelled on average further west and further away from the breeding beaches than females, both sexes exhibited a great overlap in their respective foraging location. Furthermore, males and females, which were tracked in different years, remained in the vicinity of the breeding island: on average <150 km the first month, <360 km the first three months, and foraged both over and beyond the continental shelf during that period^41^. In contrast, in the present study, the abrupt drop in δ^13^C values observed at weaning (drop of 3.7 and 2.8‰ in males and females, respectively) suggests that all sampled individuals left the Kerguelen shelf at weaning, foraged south of the Polar Front, in the Antarctic Zone, and that males travelled longer distances and foraged further south than females.

Such an early development of sexual niche differentiation is a surprising and unusual pattern. Studies across various taxa have shown that, in most cases, when sexual segregation occurs between adults, male and female juveniles share the same ecological niche e.g.^7^,^42^,^43^,^44^. When the timing of niche differentiation could be identified, some studies highlighted factors likely to explain sexual segregation in adults, such as body size^32^,^45^, growth rate^3^, or social interactions (behavioural dominance hypothesis^9^,^46^). Why then, at Kerguelen, do male and female Antarctic fur seals segregate as soon as they wean? During lactation, mothers of males and females exploited a similar isotopic niche and weaned pups forage independently from their mother^47^, thus excluding vertical transmission hypotheses.

It is possible that small size differences at weaning and/or variation in behaviour between males and females could enable male YOY to exclude female YOY from their foraging grounds. Alternatively, niche segregation in weaned pups could be the result of differential nutritional needs between the two sexes. Indeed, Antarctic fur seal pups are known to already diverge in the way they utilise the energy delivered by their mother during lactation^48^. While there is no difference in maternal investment depending on the sex of the offspring (in terms of milk composition or amount of delivered milk), male and female pups differ in their body composition at weaning. Females accumulate greater body lipid reserves than males which direct more energy into lean tissue growth^48^. Hence, it is likely that after lactation, males would continue to favour growth and target protein rich prey, while females would favour survival during this high mortality post-weaning phase and target more lipid rich prey. Accordingly, while the Antarctic krill seems to be a beneficial prey due to its high abundance in Antarctic waters, it has low lipid content: 2 to 6% of wet mass^50^, which contrasts with the high lipid content of the main myctophid fish species eaten by breeding females in Kerguelen: *Gymnoscopelus nicholsi*: 18% of wet mass, *G. fraseri*: 12%, *Electrona subaspera*: 9%^49^.

Males and females might also vary in their respective post-weaning dispersal behaviour. Indeed, males might benefit from exploring a greater diversity of foraging grounds while a more profitable strategy for females would be to rapidly gain knowledge on the local area by staying closer to the breeding colony. Accordingly, the sampled males exploited an isotopic niche five times larger than females and exhibited more inter-individual variation. Hence, it is likely that males and females differ in their dietary and dispersal benefits in preparation for future requirements, resulting in an early stage sexual segregation. While it is often difficult to distinguish between innate and learned behaviour^51^, the abruptness and consistency of δ^13^C and δ^15^N drops at weaning, in every sampled individuals, suggest that early life foraging is an innate behaviour and that sexual segregation is caused by differential innate resource preferences by the two sexes. While the ultimate drivers of this segregation still need to be investigated, the present study reveals an early life segregation that is mostly decoupled from size dimorphism and breeding constraints.

## Methods

All experimental protocols were approved by the French Polar Institute IPEV ethic committee and the methods were carried out in accordance with the approved guidelines.

### Fieldwork and isotopic analysis

The study was conducted during the 2013 mating season (November–December), at the Pointe Suzanne breeding colony (49°26’S, 70°26’E), Kerguelen Archipelago, which is located north of the Polar Front in the southern Indian Ocean. Eight “young” fur seals of each sex were captured using a hoop net and anaesthetised using isoflurane delivered via a portable gas vaporizer. Seals were of unknown age except for one 5-year-old male tagged as a pup. The approximate age of individuals was estimated in the field based on seals’ size and behaviour. Males were not breeders, while all but one female were provisioning a pup. The longest whisker was collected in each individual by cutting it as close to the skin as possible. A whisker from five additional breeding females and five adult breeding males holding a territory were sampled in order to describe sexual segregation in full-size breeding adults; and compare the isotopic niche of younger seals throughout their development with the niche of adults (during the same year, *i.e.* same environmental conditions). Females were captured and anaesthetised as previously described. Territorial bulls were manually restrained using a hoop net before being sedated by intramuscular injection of a tiletamine-zolazepam mixture (Zoletil, Virbac, France, 0.75 mg/kg estimated weight).

In the laboratory, whiskers were hand-washed in 100% ethanol and cleaned in distilled water for 5 minutes in an ultrasonic bath. They were dried, measured and cut into 3 mm-long consecutive sections starting from the proximal (facial) end. The δ^13^C and δ^15^N values of each whisker section were determined by a continuous flow mass spectrometer (Thermo Scientific, Delta V Advantage) coupled to an elemental analyser (Thermo Scientific, Flash EA 1112). Results are presented in the conventional δ notation relative to Vienna PeeDee Belemnite marine fossil limestone and atmospheric N_2_ for δ^13^C and δ^15^N, respectively. Replicate measurements of internal laboratory standards (acetanilide) indicated measurement errors <0.10‰ for both δ^13^C and δ^15^N.

### Age determination

Studies on subantarctic and South American fur seals have previously shown that three stages could potentially be identified in the whiskers of young seals: the lactation, weaning and post-weaning periods^11^,^17^. During the lactation period, females mobilise their own tissues to synthesise milk, such that their pup appears to feed at a higher trophic level than their mother and exhibit high δ^15^N values^52^. Weaning is then characterised by an abrupt drop in δ^15^N values, which is followed by the post-weaning period when the pup forages by itself and its δ^15^N signature reflects its diet^10^,^53^. Hence, in the present study, high δ^15^N values in the tip (*i.e.* oldest part) of the whisker, followed by an abrupt drop were considered to correspond to the lactation and weaning periods, respectively, and an indication that the whisker recorded the whole life of the individual. δ^15^N drop was also used as a temporal marker to age and align whiskers as it corresponds to a known event in seals life.

In some species of otariids, whiskers exhibit a consistent periodicity of δ^13^C and/or δ^15^N values along their length, corresponding to the annual cycle of seals^16^,^19^,^20^,^54^. The periodicity of δ^13^C and δ^15^N values of young and adult seals was assessed, using the wavelet analysis following Kernaléguen, *et al*.^11^. This analysis allowed us to detect: (i) if the isotopic signature of whiskers consist of a repeated periodic signal; and (ii) if the period of the cyclic pattern is consistent along the length of the whisker^55^,^56^. The assumptions of a constant growth rate and that isotopic cycles (of consistent period) correspond to annual breeding cycles were cross-validated by comparing the age determined by the wavelet analysis (i.e. number of cycles of consistent period) and the actual age of the 5-year-old tagged male.

### Comparison of isotopic niches

The temporal variation of the isotopic niche was investigated (i) for a given sex, i.e. evolution of an individual’s niche as it ages (males and females separately), and (ii) between the sexes, i.e. comparison of males and females of the same age, during the same years (i.e. same environmental conditions). The isotopic niche of each individual was determined for each age-class: from the end of weaning until the seal is one (age 0, when seals are called young of the year (YOY)), from age one to two (age 1), and so on until age 4. The isotopic values of full-size territorial bulls and breeding females were also analysed, as a control. Only the isotopic values corresponding to the last, most recent year were considered as the breeding status of the sampled adult seals was unknown during the previous years.

Niche segregation was estimated as the percentage of niche overlap and variation in niche size between two age-classes/sexes. These two parameters were calculated on the 2 dimensions of the isotopic niche (δ^13^C and δ^15^N combined) using the Bayesian ellipse-based metrics SIBER Stable Isotope Bayesian Ellipses in R^21^, using the SIAR package in R^21^. The standard ellipse area corrected for unbalanced sample sizes (SEAc) provides an estimation of the core isotopic niche of a group (equivalent of standard deviation for bivariate data) and was the metric used to calculate the percentage of niche overlap between two groups. Variation in SEAc size was statistically estimated using 10^4^ posteriori draws. SEAc were estimated at the population level, meaning that all male or female data were pooled together. However, whisker isotopic signature provides multiple values per individual that are not independent. Hence, SEAc results should only be interpreted in combination with results from the mixed effect models.

Segregation can occur either along the δ^13^C axis, the δ^15^N axis, or both axes together. Hence, comparison of δ^13^C and δ^15^N values were also performed separately. Mixed effect models were used in order to account for the repeated measurements per individual and the time-correlation of the data. The effects of sex/age (fixed effect) and individual (random effect) on δ^13^C and δ^15^N values were tested for each age-class/sex based on models’ Akaike Information Criteria (AIC) and Akaike weigth (ωAIC) which represents the relative likelihood of candidate models, after checking the residuals were normally distributed and the variance were homogenous across the fitted values. All results are presented as mean ± SD, and results were considered significant at the P < 0.05 level. All statistics were performed using R 3.0.3.

## Additional Information

**How to cite this article**: Kernaléguen, L. *et al*. Early-life sexual segregation: ontogeny of isotopic niche differentiation in the Antarctic fur seal. *Sci. Rep.* **6**, 33211; doi: 10.1038/srep33211 (2016).

## Supplementary Material

Supplementary Information

## Figures and Tables

**Table 1:** Sexual variation in two-dimension isotopic niche, at each age-class.

Age-class	SEAc Overlap (%)		SEAc size (‰^2^)		
	Male	Female	Male	Female	P value
Age 0	0	0	2.8	0.5	<0.001
Age 1	18	29	2.2	1.4	0.01
Age 2	0	0	1.2	1.2	0.41
Age 3	0	0	1.5	1.7	0.21
Age 4	0	0	2.2	0.5	<0.001
Adults	0	0	5.8	1.3	<0.001

Sexual segregation was estimated as the percentage of overlap between males’ and females’ SEAc and variation in SEAc size.

**Table 2:** Sexual variation in whisker δ^13^C and δ^15^N values at each age-class.

Age-class	Male (‰)	Female (‰)	AIC		ωAIC	
			~1	~Sex	~1	~Sex
δ^13^C						
Age 0	−22.2 ± 1.3	−21.0 ± 0.8	151.3	147.6	0.14	**0.86**
Age 1	−21.3 ± 1.5	−19.8 ± 0.9	259.6	254.5	0.07	**0.93**
Age 2	−21.2 ± 1.3	−19.6 ± 0.9	193.6	187.2	0.04	**0.96**
Age 3	−20.9 ± 1.0	−19.2 ± 0.7	227.3	215.5	0.01	**0.99**
Age 4	−21.7 ± 0.8	−18.8 ± 0.4	190.5	163.7	0.01	**0.99**
Adult	−21.2 ± 1.5	−18.9 ± 0.4	308.0	299.1	0.01	**0.99**
δ^15^N						
Age 0	9.0 ± 0.9	10.2 ± 0.4	78.8	71.6	0.03	**0.97**
Age 1	9.6 ± 0.8	10.3 ± 0.4	198.5	195.6	0.19	**0.81**
Age 2	10.1 ± 0.7	10.2 ± 0.4	174.5	176.4	**0.73**	0.27
Age 3	10.6 ± 0.6	10.4 ± 0.4	203.6	204.5	**0.61**	0.39
Age 4	10.2 ± 0.7	10.5 ± 0.3	238.4	238.6	**0.53**	0.47
Adult	11.0 ± 1.1	10.6 ± 0.4	299.8	300.5	**0.58**	0.42

The effect of sex on δ^13^C and δ^15^N values was tested for each age-class using mixed effect models to account for the repeated measurements for each individual (random effect) and the time-correlation of the data (auto-correlation coefficient). The most parsimonious models have been chosen according to their relative Akaike weight and are indicated in bold.
