# Supplementary material for: Early-life sexual segregation: ontogeny of isotopic niche differentiation in the Antarctic fur seal
Source: Sci Rep. 2016 Sep 13;6:33211. doi: 10.1038/srep33211 (PMC5020412; doi:10.1038/srep33211)
Supplement: Supplementary Information [file srep33211-s1.doc]

**Early life sexual segregation: ontogeny of isotopic niche differentiation in the Antarctic fur seal**

L. Kernaléguen, J. P. Y. Arnould, C. Guinet, B. Cazelles, P. Richard and Y. Cherel

**Supplementary Information**


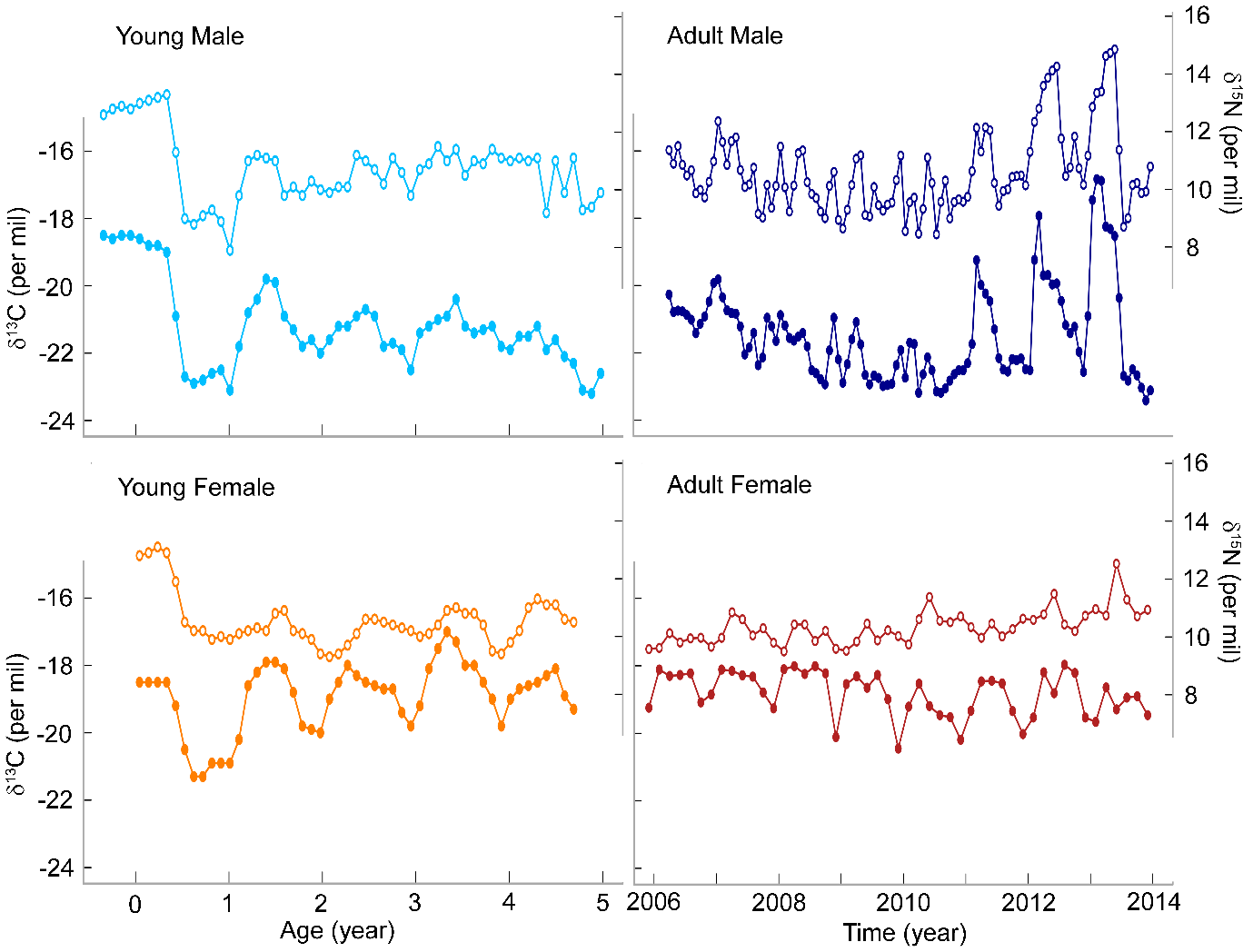


**Supplementary Fig. S1. Whisker δ^13^C (filled circles) and δ^15^N (open circles) values of one representative young and full-size breeding male and female.** All young individuals presented high δ^15^N values in the tip (i.e. oldest part displayed on the left) of the whisker, followed by an abrupt drop of both δ^13^C and δ^15^N values, corresponding to the lactation and weaning periods, respectively. The young male was a tagged 5-year-old seal.

**Supplementary Table S1. Ontogenetic changes in females’ isotopic niche during the first 5 years of life.** Niche variation was estimated by the niche overlap (percentage of SEAc overlap, down left values) and variation in niche size (SEAc size in brackets (‰^2^), with top right values presenting the P value corresponding of the comparison of SEAc sizes) using the Bayesian ellipse-based metrics SIBER. Overlap values are given as the percentage of overlap over the total niche area of a given age-class. The first value is the overlap of the youngest age-class over the oldest, and the second value corresponds to the overlap of the oldest age-class over the youngest.

|  | Age 0 (0.5) | Age 1 (1.4) | Age 2 (1.2) | Age 3 (1.7) | Age 4 (0.5) | Adult (1.3) |
| --- | --- | --- | --- | --- | --- | --- |
| Age 0 (0.5) |  | 0.04 | 0.15 | 0.01 | 0.13 | 0.10 |
| Age 1 (1.4) | 0 / 0 |  | 0.18 | 0.20 | <0.001 | 0.29 |
| Age 2 (1.2) | 0 / 0 | 55 / 67 |  | 0.04 | 0.01 | 0.36 |
| Age 3 (1.7) | 0 / 0 | 71 / 57 | 87 / 57 |  | <0.001 | 0.09 |
| Age 4 (0.5) | 0 / 0 | 21 / 57 | 27 / 61 | 29 / 98 |  | <0.001 |
| Adult (1.3) | 0 / 0 | 46 / 51 | 58 / 53 | 65 / 90 | 92 / 37 |  |

**Supplementary Table S2. Age-dependent changes in whisker δ^13^C and δ^15^N values of females. The effect of age-class on δ^13^C and δ^15^N values was tested using mixed effect models to account for the repeated measurements for each individual (random effect) and the time-correlation of the data (auto-correlation coefficient). The most parsimonious models have been chosen according to their relative Akaike weight.**

|  |  | AIC | | ωAIC | |
| --- | --- | --- | --- | --- | --- |
|  |  | ~ 1 | ~ Age | ~ 1 | ~ Age |
| δ^13^C (‰) | | 563.9 | 506.6 | <0.0001 | 1 |
| δ^15^N (‰) | | 416.9 | 425.5 | 0.99 | 0.01 |
| δ^13^C (‰) | Age 0  (-20.9 ± 0.3) | Age 1  (-19.4 ± 0.5) | Age 2  (-19.1 ± 0.4) | Age 3  (-18.9 ± 0.4) | Age 4  (-18.6 ± 0.4) |
| Age 0 (-20.9 ± 0.3) |  |  |  |  |  |
| Age 1 (-19.4 ± 0.5) | t_222,222_=7.69 (<0.001) |  |  |  |  |
| Age 2 (-19.1 ± 0.4) | t_222,222_=9.80 (<0.001) | t_222,222_=1.41 (0.16) |  |  |  |
| Age 3 (-18.9 ± 0.4) | t_222,222_=10.29 (<0.001) | t_222,222_=2.56 (0.01) | t_222,222_=1.41 (0.16) |  |  |
| Age 4 (-18.6 ± 0.4) | t_222,222_=14.15 (<0.001) | t_222,222_=4.90 (<0.001) | t_222,222_=3.98 (<0.001) | t_222,222_=1.75 (0.08) |  |
| Adult (-18.8 ± 0.5) | t_222,8_=9.58 (<0.001) | t_222,8_=2.60 (0.03) | t_222,8_=1.58 (0.15) | t_222,8_=0.32 (0.75) | t_222,8_=-1.06 (0.32) |
| δ^15^N (‰) | Age 0  (10.3 ± 0.4) | Age 1  (10.5 ± 0.3) | Age 2  (10.3 ± 0.3) | Age 3  (10.6 ± 0.3) | Age 4  (10.6 ± 0.3) |
| Age 0 (10.3 ± 0.4) |  |  |  |  |  |
| Age 1 (10.5 ± 0.3) | t_222,222_=1.42 (0.16) |  |  |  |  |
| Age 2 (10.3 ± 0.3) | t_222,222_=0.29 (0.77) | t_222,222_=-1.56 (0.12) |  |  |  |
| Age 3 (10.6 ± 0.3) | t_222,222_=1.91 (0.06) | t_222,222_=0.66 (0.51) | t_222,222_=2.18 (0.03) |  |  |
| Age 4 (10.6 ± 0.3) | t_222,222_=2.51 (0.01) | t_222,222_=1.41 (0.16) | t_222,222_=2.99 (0.003) | t_222,222_=0.72 (0.47) |  |
| Adult (10.7 ± 0.6) | t_222,8_=1.39 (0.20) | t_222,8_=0.68 (0.52) | t_222,8_=1.33 (0.22) | t_222,8_=0.38 (0.71) | t_222,8_=0.05 (0.96) |

**Supplementary Table S3. Ontogenetic changes in males’ isotopic niche during the first 5 years of life.** Niche variation was estimated by the niche overlap (percentage of SEAc overlap, down left values) and variation in niche size (SEAc size in brackets (‰^2^), with top right values presenting the P value corresponding of the comparison of SEAc sizes) using the Bayesian ellipse-based metrics SIBER. Overlap values are given as the percentage of overlap over the total niche area of a given class-age. The first value is the overlap of the youngest age-class over the oldest, and the second value corresponds to the overlap of the oldest age-class over the youngest.

|  | Age 0 (2.8) | Age 1 (2.2) | Age 2 (1.2) | Age 3 (1.5) | Age 4 (2.2) | Adult (5.8) |
| --- | --- | --- | --- | --- | --- | --- |
| Age 0 (2.8) |  | 0.16 | <0.001 | <0.001 | 0.14 | <0.001 |
| Age 1 (2.2) | 32 / 40 |  | <0.001 | 0.01 | 0.45 | <0.001 |
| Age 2 (1.2) | 3 / 7 | 30 / 54 |  | 0.14 | <0.001 | <0.001 |
| Age 3 (1.5) | 0 / 0 | 14 / 20 | 69 / 56 |  | 0.01 | <0.001 |
| Age 4 (2.2) | 17 / 21 | 14 / 14 | 35 / 19 | 49 / 34 |  | <0.001 |
| Adult (5.8) | 0 / 0 | 2 / 1 | 41 / 9 | 75 / 19 | 74 / 28 |  |

**Supplementary Table S4. Age-dependent changes in whisker** **δ^13^C and δ^15^N values of males.** The effect of age-class on δ^13^C and δ^15^N values was tested using mixed effect models to account for the repeated measurements for each individual (random effect) and the time-correlation of the data (auto-correlation coefficient). The most parsimonious models have been chosen according to their relative Akaike weight.

|  |  | | AIC | | | | ωAIC | | | |
| --- | --- | --- | --- | --- | --- | --- | --- | --- | --- | --- |
|  |  | | ~ 1 | | ~ Age | | ~ 1 | | ~ Age | |
| δ^13^C (‰) | | | 1072.0 | | 1008.9 | | <0.0001 | | 1 | |
| δ^15^N (‰) | | | 930.2 | | 846.2 | | <0.0001 | | 1 | |
| δ^13^C (‰) | Age 0  (-22.2 ± 0.7) | Age 1  (-21.0 ± 0.7) | | Age 2  (-20.9 ± 0.6) | | Age 3  (-20.9 ± 0.6) | | Age 4  (-21.8 ± 0.6) | |  |
| Age 0 (-22.2 ± 0.7) |  |  | |  | |  | |  | |  |
| Age 1 (-21.0 ± 0.7) | t_379,379_=5.05 (<0.001) |  | |  | |  | |  | |  |
| Age 2 (-20.9 ± 0.6) | t_379,379_=5.94 (<0.001) | t_379,379_=0.42 (0.68) | |  | |  | |  | |  |
| Age 3 (-20.9 ± 0.6) | t_379,379_=5.77 (<0.001) | t_379,379_=0.38 (0.70) | | t_379,379_=-0.02 (0.98) | |  | |  | |  |
| Age 4 (-21.8 ± 0.6) | t_379,379_=1.59 (0.11) | t_379,379_=-4.55 (<0.001) | | t_379,379_=-6.02 (<0.001) | | t_379,379_=-5.70 (<0.001) | |  | |  |
| Adult (-21.1 ± 0.9) | t_379,379_=2.89 (0.02) | t_379,379_=-0.25 (0.81) | | t_379,379_=-0.45 (0.66) | | t_379,379_=-0.43 (0.67) | | t_379,379_=2.09 (0.06) | |  |
| δ^15^N (‰) | Age 0  (9.0 ± 0.4) | Age 1  (9.8 ± 0.4) | | Age 2  (10.3 ± 0.4) | | Age 3  (10.6 ± 0.4) | | Age 4  (10.1 ± 0.4) | |  |
| Age 0 (9.0 ± 0.4) |  |  | |  | |  | |  | |  |
| Age 1 (9.8 ± 0.4) | t_379,379_=6.00 (<0.001) |  | |  | |  | |  | |  |
| Age 2 (10.3 ± 0.4) | t_379,379_=9.93 (<0.001) | t_379,379_=4.57 (<0.001) | |  | |  | |  | |  |
| Age 3 (10.6 ± 0.4) | t_379,379_=12.22 (<0.001) | t_379,379_=7.41 (<0.001) | | t_379,379_=3.09 (0.002) | |  | |  | |  |
| Age 4 (10.1 ± 0.4) | t_379,379_=6.82 (<0.001) | t_379,379_=2.01 (0.04) | | t_379,379_=-1.42 (0.16) | | t_379,379_=-3.64 (<0.001) | |  | |  |
| Adult (11.1 ± 0.6) | t_379,10_=7.59 (<0.001) | t_379,10_=4.83 (<0.001) | | t_379,10_=3.06 (0.01) | | t_379,10_=1.90 (0.09) | | t_379,10_=3.57 (0.005) | |  |
